# The world’s oldest living culture leads the way with interpretable generative AI: Aboriginal women shaping the future of perinatal mental health services

**DOI:** 10.1371/journal.pdig.0001709

**Published:** 2026-10-15

**Authors:** Guanjin Wang, Hachem Bennamoun, Wai Hang Kwok, Rehan Raza, Ferdous Sohel, Bridgette Kelly, Trish Ratajczak, Corinne Reid, Rhonda Marriott, Roz Walker, Jayne Kotz, Tracy Reibel

**Affiliations:** 1 School of Information Technology, Murdoch University, Perth, Western Australia, Australia; 2 Ngangk Yira Institute for Change, Murdoch University, Perth, Western Australia, Australia; 3 School of Nursing and Midwifery, Edith Cowan University, Perth, Western Australia, Australia; 4 Global Health Academy and Academy of Sport, The University of Edinburgh, Edinburgh, United Kingdom; Australian Institute of Health Innovation, AUSTRALIA

## Abstract

Despite their strengths, Aboriginal mothers experience significant health disparities, including perinatal mental health challenges influenced by social, historical, and cultural determinants. Routine risk screening informs follow-up care, yet conventional approaches often fail to address cultural considerations. The Baby Coming You Ready (BCYR) program provides a strengths-based, culturally adapted digital assessment designed to enhance perinatal mental health care. However, some clinicians continue to rely primarily on conventional risk scores, which can neglect broader contextual circumstances influencing Aboriginal mothers, including cultural strengths and protective factors, thereby limiting the program’s full impact. This exploratory model-development study examined whether generative AI data augmentation combined with an intrinsically interpretable machine-learning model could support culturally contextualised prediction of perinatal psychological distress among Aboriginal mothers. We analysed deidentified BCYR pilot data from 293 Aboriginal mothers across six Western Australian sites. After excluding five records with incomplete outcome data, the analytic dataset included 288 participants. Models were evaluated using repeated internal train-test splits, with synthetic data used only for training augmentation and performance assessed on held-out real test data. The highest internal performance was observed for the TVAE-augmented Explainable Boosting Machine at an augmentation ratio of 1.0, with an accuracy of 0.867, an F1 score of 0.817, and an AUC of 0.934. Model explanations identified self-blame, loneliness, family pride, day-to-day management, sleep, and related contextual factors as influential contributors to model-estimated risk. These explanations are intended as prompts for culturally safe discussion and clinical reflection. Findings suggest that interpretable generative AI may have potential to support culturally responsive perinatal mental health assessment. However, broader community-governed external validation and prospective clinical workflow evaluation are required before clinical implementation.

## Author summary

Perinatal mental health care for Aboriginal mothers should recognise their strengths, cultural context, family relationships, and lived experiences. Standard screening tools may overlook these important factors and may not support culturally safe conversations between Aboriginal mothers and health professionals. The Baby Coming You Ready program was developed to provide a strengths-based, culturally adapted digital assessment for Aboriginal families. However, the contextual and strengths-based information captured through BCYR may not always be fully incorporated into clinical interpretation. This exploratory model-development study examined whether generative and interpretable AI could support culturally contextualised prediction of perinatal psychological distress among Aboriginal mothers. We tested whether carefully augmented training data could support model development while retaining real participant data as the analysis foundation. We also used an intrinsically interpretable model so that the contributing factors could be examined. The best-performing model identified factors such as self-blame, loneliness, family pride, day-to-day management, sleep, and related contextual factors as important contributors to model-estimated psychological distress. The model explanations are intended as prompts for culturally safe discussion and clinical reflection, rather than to replace clinical judgement. However, this study remains exploratory, and further Aboriginal-led co-design, external validation, fairness assessment, and real-world evaluation are needed before clinical implementation.

## 1 Introduction

Perinatal depression and anxiety affect maternal and infant health and well-being. Studies show that maternal depression and anxiety are associated with adverse infant outcomes such as preterm birth and stillbirths [1], intrauterine growth restriction [2], low birth weight, disrupted maternal/infant bonding, substance misuse, infant removal [3], and maternal suicide [4,5]. Many Aboriginal Australian women are strong, resilient mothers. However, at a population level, many experience increased risks of worsening depression or anxiety due to sustained impacts of colonisation, cultural disruption, and policies until the 1970s that led to the systematic removal of Aboriginal children from their families, referred to as the Stolen Generations [6]. Other factors include racism, poverty, trauma, prejudice, and the widening inequities in social determinants [7,8]. A recent systematic review found Aboriginal women experience a 38% higher likelihood of depression, a 79% higher chance of mental health problems in pregnancy, and a 30% higher chance after birth, compared to their non-Aboriginal counterparts [9]. A previous Western Australian study found that the mothers of one in three Aboriginal babies born between 1997 and 2013 sought hospital care for perinatal mental health problems [10]. Also, Mah et al. [11] reported extremely high rates of psychological distress among pregnant Indigenous Australian women living in rural and remote communities in NSW, with 36.4% scoring above the standard cut-off for distress. Despite more than two decades of routine perinatal mental health screening in Australia, significant disparities in health and well-being persist between Aboriginal mothers and infants and their non-Aboriginal counterparts. These inequities are reflected in significantly higher rates of preterm births, stillbirths, low birth weight infants, and infant removals among Aboriginal families [7,8]. Recent research has found that conventional perinatal healthcare is often culturally unsafe and frequently retraumatising for Aboriginal women [12]. Aboriginal women have reported feeling judged and fearful when engaging with current risk screening tools due to cultural unsafety, misalignment between medicalised assessment approaches and lived experience, and ongoing concerns related to child removal, and thus alienated from potentially supportive care [13,14].

The Baby Coming You Ready (BCYR) program, co-designed to address these challenges, is actively guided by a panel of Indigenous Elders, healthcare professionals, and Indigenous midwives. The program is built around a digitised, strengths-based, culturally safe, and holistic perinatal assessment/screen, which incorporates all seven elements of Aboriginal social and emotional wellbeing [15]. The assessment/screen uses touchscreen images on iPads with Aboriginal voice-overs guiding the self-reflective inquiry. Aboriginal mothers select images they relate to. This visually promotes engagement and self-reflection, guiding mothers to prioritize their strengths and worries and then self-determine relevant solutions. Between 2021 and 2023, BCYR was piloted in WA perinatal health-care sites, serving as an alternative to existing screening tools for mental health, family and domestic violence (FDV), and alcohol, tobacco, and other drugs (AOD) use. However, some midwives and service managers expressed uncertainty about culturally safe clinical reasoning and decision-making, often relying on single risk-screen scores to guide their clinical responses and referrals [14]. Such resistance may undermine the promising potential of the BCYR assessment to empower Aboriginal women in self-identifying and self-determining their perinatal health care needs. This underscores the need for approaches that more effectively support culturally responsive and strengths-based assessment practices.

To address these identified gaps in Aboriginal-focused perinatal mental health screening and care, we aimed to develop and internally evaluate a visual, culturally respectful, strengths-based clinical decision-support model that integrates BCYR assessment data with generative AI and explainable AI (XAI) technologies. Specifically, to examine whether internal model performance and explanation quality could be improved under limited-data conditions, this approach leverages generative AI to augment the deidentified holistic and culturally contextualised assessment data from the BCYR platform, and uses XAI techniques to clarify the rationale behind the model’s predictions. Particularly, our model enables the identification, prioritisation, and weighting of maternal risk-related and protective-related factors, providing a quantified assessment of their influence on model-estimated perinatal mental health and well-being at both individual and group levels. We also developed a prototype dashboard that visually presents model-identified protective-related and risk-related factors, integrates relevant BCYR images, and displays model-estimated outcomes to enhance accessibility. This work represents an early-stage approach to engaging AI within culturally safe healthcare practices, aligning with calls from recent studies such as [16] to integrate AI into Indigenous knowledge frameworks and to develop tools that are meaningful and relevant to communities themselves. The dashboard requires further Aboriginal-led co-design and usability evaluation to determine whether it supports self-determination, cultural safety, and respectful models of care in practice. If validated in future studies, such a tool may assist health professionals to better understand culturally contextualised model outputs and use them as prompts for holistic, woman-centered conversations. However, whether this approach improves clinical confidence, trust, engagement, empowerment, or outcomes remains an empirical question requiring prospective evaluation.

It is important to distinguish the technological contribution of this study from clinical validation. This work should be understood as an exploratory model-development and internal-validation study using deidentified BCYR pilot data. The aim is to examine whether generative AI augmentation and an intrinsically interpretable model can improve internal predictive performance and provide culturally meaningful explanations. The model is not presented as a diagnostic tool, a replacement for clinical judgment, or a validated basis for referral or service allocation. Before any clinical implementation, the model and dashboard require independent external validation, prospective testing in real-world care pathways, evaluation of potential harms and bias, and ongoing Aboriginal community governance.

The remainder of this paper is organised as follows. Section 2 summarises relevant research, including generative AI and XAI techniques. Section 3 describes the dataset adopted and the methodology design. Section 4 presents results and discussions. Section 7 provides the conclusion and suggests future work.

## 2 Related work

### 2.1 Generative AI for data augmentation

Building effective and robust healthcare prediction models requires large, high-quality datasets for training, which can be particularly challenging for underrepresented populations. For example, Aboriginal and Torres Strait Islander peoples comprise approximately 3.3% of the Western Australian population [17], and this small population size, combined with privacy concerns, data collection costs, and the rarity of certain conditions [18,19], could limit the availability of data needed to develop effective population-specific prediction models [20]. At the same time, small datasets in Aboriginal community-based health research should not be understood only as a technical limitation. They may also reflect locally distinct and context-specific communities. Therefore, this study explores whether locally grounded modelling can support more context-sensitive prediction within specific service and community settings.

To address data scarcity, generative AI techniques, known for their potential to generate synthetic data instances similar to training data, have shown promise in this area [21]. By supplementing small datasets with carefully evaluated synthetic data, researchers may improve model robustness in internal experiments. However, synthetic data does not replace the need for real-world diversity, external validation, or appropriate consent and governance.

Many studies have explored generative AI-based methods for augmenting tabular data while preserving the statistical properties and relationships present in the original data. For instance, Ahmed et al. [22] employed various GAN-based methods to generate high-quality synthetic tabular healthcare data for cancer detection. In their evaluation, CGAN and CTGAN more effectively maintained inter-feature relationships while improving model predictive performance on synthetic and hybrid datasets. Similarly, Liu et al. [23] utilized four different generative models, including CTGAN, Bayesian network, sequential decision tree, and TVAE, to generate synthetic tabular health data for addressing the challenges of small clinical data in prognostic tasks. Anshelevich and Katz [24] introduced a hierarchical framework, T-VAE-GAN, in which a VAE first learns the data distribution and guides a downstream GAN to generate high-quality tabular synthetic samples. Saadatinia and Salimi-Badr [25] introduced an explainable CNN-based method for schizophrenia mental health diagnosis, and generated synthetic data using VAE and Wasserstein GAN with gradient penalty (WGAN-GP) to address the overfitting issue in limited datasets. Their results showed that VAE-based augmentation led to faster convergence and high performance. Similarly, Li et al. [26] proposed a tabular autoencoder GAN (TAEGAN) that integrates a masked AE as a generator, generating realistic tabular augmented data to address class imbalance and limited data issues in healthcare.

Inspired by recent advances in VAE and GAN-based data augmentation methods for healthcare and mental health analytics, this study utilises CTGAN and TVAE to generate synthetic training data from a limited dataset, addressing data scarcity within an exploratory internal-validation setting. Gaussian Copula was also included as a baseline statistical synthesizer. The following subsections provide a brief review of these techniques for data augmentation, with a focus on their specific adaptations for tabular data.

#### 2.1.1 GAN and CTGAN.

The Generative Adversarial Networks (GANs) framework creates generative models by employing a two-network adversarial process [27–29]. One network (the generator or *G*) learns the underlying data distribution of the training set and produces synthetic samples intended to resemble real data. A second network (the discriminator or *D*) is tasked with distinguishing between real and generated samples.

The discriminator iteratively improves its capacity to differentiate between real from synthetic data by estimating the probability that a given input originates from the training data rather than from *G*. The generator, in turn, seeks to maximize the discriminator’s error rate by generating increasingly realistic data. This adversarial interaction creates a minimax game dynamic. GAN networks are trained using backpropagation, which allows the generative model to improve without relying on more complex methods like Markov chains or approximate inference networks. This results in a generative model capable of producing high-quality samples that closely resemble the original training data. The objective function of GAN is defined as follows:


$$\begin{array}{c} \min_{G}\max_{D}V(D,G)\;=\;{𝔼}_{x\sim p_{\text{data}}(x)}[\log D(x)]\;+\;{𝔼}_{z\sim p_{z}(z)}\left[\log\left(1-D\left(G(z)\right)\right)\right]\; \end{array}$$ (1)

where *x* represents real data samples from the real data distribution $p_{data}(x)$, and *z* represents input noise variables sampled from a prior distribution $p_{z}(z)$ [29,30]. *G*(*z*) is the generative model that maps the noise variable *z* to the data space, generating fake samples. *D*(*x*) is the discriminative model that outputs the probability that *x* is a real data sample.

Conditional Tabular GAN (CTGAN) is a variant of GAN specifically designed for generating synthetic tabular data [30]. It combines the power of conditional generative adversarial networks (cGANs) with the ability to handle mixed data types (continuous and categorical) commonly found in tabular datasets. The objective function of CTGAN is presented below:


$$\begin{array}{cc} \min_{G}\max_{D}V(D,G)=\; & {𝔼}_{x\sim p_{\text{data}}(x)}[\log D(x|c)]\\ & +\;{𝔼}_{z\sim p_{z}(z),\;c\sim p_{\text{cond}}(c)}[\log(1-D(G(z|c)|c))] \end{array}$$ (2)

where *c* is the conditioning variable sampled from the conditional distribution *p*_cond_(*c*), and G(z∣c) is the generated sample from the generator *G* conditioned on *c*.

#### 2.1.2 VAE and TVAE.

The Variational Autoencoders (VAEs) network [31] is a generative model for efficient inference and for learning a probabilistic mapping from a high-dimensional input space to a lower-dimensional latent space, and then map back from the latent space to the input space. The architecture consists of an encoder network (*E*) and a decoder network (*D*), where the encoder projects the input data into a low-dimensional latent space, while the decoder reconstructs the input from this latent representation. Essentially, the encoder effectively compresses the input while the decoder attempts to reconstruct it, thereby enabling the model to learn compact, meaningful representations of the data. The primary goal of VAE is to generate new, similar data samples by drawing from the learned latent distribution. The objective function of VAE is to maximize the evidence lower bound (ELBO), consisting of reconstruction loss and Kullback-Leibler (KL) divergence loss [31].

The TVAE is based on the traditional VAE framework but is specifically designed for handling tabular data [30,31]. TVAE can handle mixed data types by employing type-specific likelihood functions in the decoder. The ELBO for TVAE for mixed data types can be expressed by combining type-specific reconstruction losses with a KL divergence term as below:


$$\begin{array}{cc} {ℒ}_{\text{TVAE}}=\; & -{𝔼}_{q(z|x)}\left[\log p(x_{\text{continuous}}|z)\right]\\ & -{𝔼}_{q(z|x)}\left[\log p(x_{\text{categorical}}|z)\right]\\ & -D_{\text{KL}}\left(q(z|x)∥p(z)\right) \end{array}$$ (3)

where *x* is the input data, *z* is the latent variable, *q*(*z*|*x*) is the approximate posterior distribution, $p_{\theta }(x|z)$ is the likelihood function, and $D_{KL}$ denotes the KL divergence loss.

### 2.2 Glassbox model for explainability

In healthcare, particularly in the context of Aboriginal perinatal mental health, clinical decision-making should be transparent, culturally responsive, and trustworthy. However, some clinicians remain hesitant to integrate AI-driven predictions into care, especially when models function as “black boxes” with limited interpretability [32,33]. XAI has emerged as a critical approach to address this challenge by providing interpretable insights into how predictions are generated, thereby supporting clinician understanding and informed decision-making [33]. Recent studies have demonstrated the potential of XAI techniques in enhancing transparency in healthcare applications [34–36].

Broadly, XAI methods are generally categorized into two groups: post hoc explanations and intrinsic explanations. Post hoc methods aim to generate explanations for black-box models that are already built; however, they may not truly reflect the internal reasoning of the model. Intrinsically interpretable models, such as decision trees and generalized additive models, are inherently designed to provide clear and interpretable outputs, and they are also simple to audit [37,38].

In this work, we adopted an inherently interpretable generalized additive model called the Explainable Boosting Machine (EBM) [39] to construct the prediction model, considering its predictive performance and its ability to provide clear explanations for its predictions across various domains [40], including healthcare [41,42]. EBM’s performance is further demonstrated through the empirical experiments presented in Section 4.

EBM is a glassbox machine learning algorithm that combines the accuracy of gradient boosting with the interpretability of generalized additive models (GAMs) [43]. It builds an ensemble of boosted decision trees, similar to gradient boosting, but constrains each tree to be a simple decision stump. This constraint allows the model to capture the main effects of each feature independently, making the model more interpretable. EBM also includes pairwise interaction terms between features, enabling it to capture complex relationships in the data. In general, EBM can be represented by the following form [44]:


$$\begin{array}{c} g(𝔼[y])=\beta_{0}+\sum_{i}f_{i}(x_{i})+\sum_{i,j}f_{ij}(x_{i},x_{j}) \end{array}$$ (4)

where g(·) denotes the link function, which adjusts the expected value of the response variable *y* depending on whether the task involves classification or regression. The term $\beta_{0}$ serves as the intercept, reflecting the predicted outcome when all input features are set to zero. The function $f_{i}(x_{i})$ represents the contribution of each individual feature to the prediction, learned through tree-based gradient boosting techniques. Similarly, $f_{ij}(x_{i},x_{j})$ captures the interaction effects between feature pairs $\left(x_{i},x_{j}\right)$, also derived from tree-based learning methods, and quantifies their joint influence on the predicted output [45].

## 3 Methodology

### 3.1 Ethics statement

Ethics approval for this study was obtained from the Human Research Ethics Committee (HREC) - Western Australia Research Governance Service (RGS000000649), Murdoch University (2021/101), and the Western Australian Aboriginal Health Ethics Committee (WAAHEC) (HREC553). Access to deidentified data was granted only for participants who provided informed consent. The participant information sheet and consent option were embedded in the digital application, allowing participants to choose whether to participate and share their deidentified data for research purposes.

The use of deidentified and synthetic data does not remove the need for Aboriginal data governance, community oversight, and culturally safe interpretation. Any future use of the model or dashboard in clinical practice will require Aboriginal-led governance, clear consent and accountability processes, and safeguards to ensure that model outputs are not used for automated, punitive, or culturally unsafe decision-making.

### 3.2 Dataset

The dataset was obtained from the BCYR program pilot study conducted in Western Australia between January 2022 and June 2023 [14]. The deidentified dataset included 293 Aboriginal mothers across six participating sites. Five records were excluded because the K5 outcome variable was incomplete, leaving a final analytic sample of 288 participants. The dataset reflects a pilot implementation sample rather than a population-based sample.

Each participant had 345 variables spanning key domains such as cultural strengths and protective factors, common life events, personal worries, family relationships, smoking, and substance use. These variables were generated based on participants’ selections of images that best represented their current circumstances at the time of screening.

The indicator of perinatal psychological distress was derived from the Kessler Psychological Distress Scale (K5), a five-item measure commonly used and validated for assessing psychological distress among Aboriginal and Torres Strait Islander populations [46].

### 3.3 Data pre-processing

Five entries were removed from the dataset due to incomplete data on the perinatal mental health indicator. The K5 was employed to assess distress levels, with each of the five items rated on a negatively keyed Likert scale from 1 to 5. Participants with a total K5 score below 12 were categorised as low risk (assigned a value of 0), whereas those scoring 12 or higher were categorised as high (elevated) risk (assigned a value of 1). After data cleaning and preprocessing, feature selection was carried out using a random forest algorithm, leveraging variable importance scores to identify influential predictors [47]. The random forest algorithm was configured with 500 trees, and the number of features randomly selected when building trees was set to the square root of the total number of input variables. An initial subset of the top 31 variables, identified based on importance scores, was selected for further review. This subset was subsequently examined by the BCYR research team and healthcare practitioners. Drawing on their domain expertise, the list was refined to a final set of 20 predictor variables, along with one output variable for prediction. As this was an exploratory model-development study, the predictor-selection process was conducted before repeated internal train-test evaluation and was informed by both data-driven ranking and domain review. Details of the selected features are presented in Table 1.

**Table 1:** Key variables for predictive model development.

CODE	FEATURE LABEL	ANSWER OPTIONS
FS1.Q225	Feeling Lonely	5: Almost always, 4: Often, 3: Sometimes, 2: A little, 1: Hardly ever
FS1.Q227	Blaming Herself	5: Almost always, 4: Often, 3: Sometimes, 2: A little, 1: Hardly ever
FS1.Q231	Life Not Worth Living	1: Never, 2: Rarely, 3: Sometimes, 4: Often, 5: Almost always
FS1.Q214	Strong Mum	5: Almost always, 4: Often, 3: Sometimes, 2: A little, 1: Hardly ever
FS1.Q562	Goal Likely	1: A lot, 2: A fair amount, 3: A little bit, 4: Not at all
FS1.Q904	Managing Day-to-Day	0: Manage well, 1: Struggle a bit, 2: Struggle a lot
FS1.Q534	Keeping Safety Plan	0: Does not agree, 1: Client agrees
FS1.Q455	Bothered by Gambling	0: Never, 1: Sometimes
FS1.Q228	Makes Family Proud	5: Almost always, 4: Often, 3: Sometimes, 2: A little, 1: Hardly ever
FS1.Q454	Family Gambles	0: No, 1: Sometimes, 2: Yes
FS1.Q664	Children in Her Care	0: 0, 1: 1, 2: 2, 3: 3, 4: 4+
FS1.Q450	Smoking Cigarettes in Pregnancy	0: No, 1: Sometimes, 2: Yes
FS1.Q661	First Pregnancy	0: No, 1: Yes
FS1.Q909	Trouble Sleeping	1: Sleeping well, 2: Trouble sleeping (not due to pregnancy/baby)
FS1.Q922	Need Help with Housing	0: No, 1: Yes
FS1.Q663	Previous Births	0: 0, 1: 1, 2: 2, 3: 3, 4: 4+
FS1.Q71	Feeling Worried	0: No, 1: Yes
FS1.Q653	Told Partner/Husband	0: No, 1: Yes
FS1.Q204	Partner Angry/Controlling	0: No, 1: Yes
FS1.Q195	Partner Moody	0: No, 1: Yes
K5	k5_classes	low risk, high risk

### 3.4 Synthetic data generation and augmentation

Conditional Tabular GAN (CTGAN) [30] and Tabular TVAE (TVAE) [30,31], implemented via the Synthetic Data Vault (SDV) [48], were utilised to create synthetic tabular datasets that emulate the statistical properties and structures of the training data. In addition, Gaussian Copula (GC), a standard statistical synthesizer in SDV, was included as a baseline.

Synthesizer hyperparameters were largely kept at default values, with minor adjustments to accommodate the limited dataset size: the embedding dimension was reduced from 128 to 64, batch size from 500 to 20, and training epochs from 300 to 30 based on observed loss convergence. CUDA acceleration was enabled to reduce computation time.

To examine the effect of synthetic data volume on internal model performance, synthetic data were generated at ratios of 0.25, 0.5, 0.75, 1, 1.25, 1.5, 1.75, and 2 relative to the real training dataset. For each ratio, five synthetic variations were generated, concatenated with the real training data, and randomly shuffled to form augmented training sets. Model performance was aggregated across the five variations for each ratio, and this process was repeated for all ratios. Within each experimental cycle, synthesizers were fitted only on the training partition. The held-out test data were not used for synthesizer fitting, data augmentation, hyperparameter tuning, model fitting, or model selection. The augmentation process is defined in Eq. (5).


$$\begin{array}{c} D_{A_{\left(r,v\right)}}=D_{T}+F(r,v,D_{T}) \end{array}$$ (5)

where $D_{T}$ refers to the original training dataset {X,𝐲} containing input features **X** and risk labels 𝐲; *r* refers to the data generation ratio selected from (0.25, 0.5, 0.75, 1, 1.25, 1.5, 1.75, 2), *v* refers to the variation index selected from 1 to 5; $F(r,v,D_{T})$ refers to the data synthesis function generating synthetic data based on *r*, *v*, and $D_{T}$; and $D_{A_{\left(r,v\right)}}$ refers to the augmented dataset $\left\{{\text{X}}_{A_{\left(r,v\right)}}^{\prime },{𝐲}_{A_{\left(r,v\right)}}^{\prime }\right\}$ for ratio *r* and variation *v*. It is important to note that the synthetic data were used only as an augmentation method, not as a replacement for real participant diversity. They were also not used to estimate prevalence, infer population distributions, or support claims of clinical generalisability. All reported model evaluations were performed on held-out, real, de-identified data.

### 3.5 Prediction model construction

We adopted the inherently interpretable Explainable Boosting Machine (EBM) [39] to construct the prediction model, given its predictive performance and ability to provide transparent explanations [40]. In addition, we employed several widely used machine learning models as baselines, which have demonstrated strong performance in prior studies, including for noisy and small tabular datasets [49]. These baseline models included Logistic Regression (LR) [50], Random Forest (RF) [51], Gradient Boosting Machine (GB) [52], and Support Vector Machine (SVM) [53].

The hyperparameters for the EBM and all baseline models were tuned using grid search or random search with stratified 10-fold cross-validation. For each experimental cycle, hyperparameter tuning was performed only within the training partition. The held-out real test set was reserved solely for final internal evaluation.

### 3.6 Model evaluation

To account for potential variability in internal model performance, the entire experimental cycle—including data splitting, generation of training and testing sets, data synthesis from the training set, augmentation of the original training data, model training on the augmented data, and evaluation on the held-out real testing set—was repeated three times using random states 3, 5, and 30. Each cycle employed different training and testing splits, and final model performance was reported as the average across the three cycles. Within each cycle, five variations of augmented data were generated for each augmentation ratio, and results were aggregated across these variations. The overall experimental workflow is illustrated in Fig 1.

**Fig 1:**
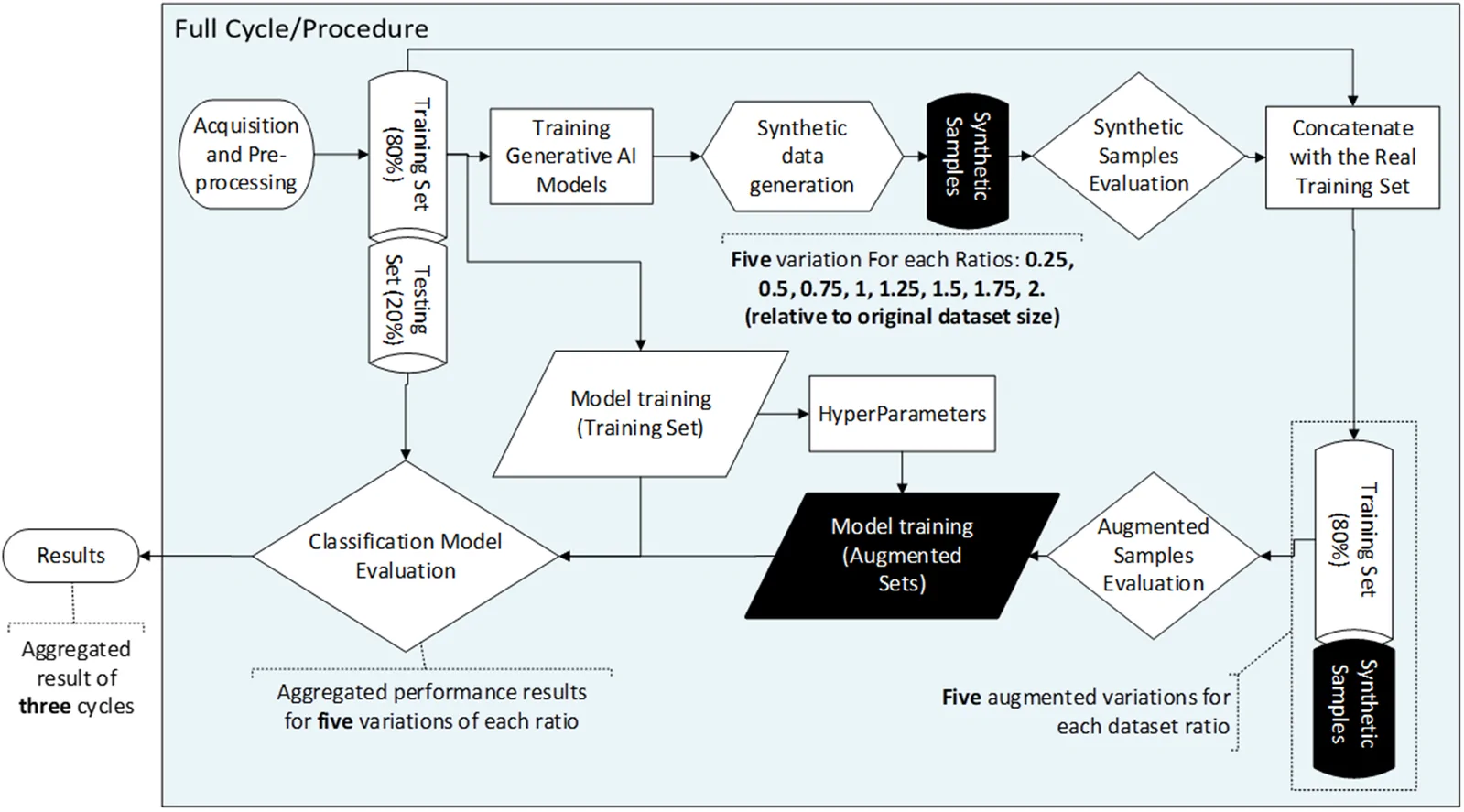
Experimental workflow for exploratory model development and internal validation. The dataset was repeatedly split into training and held-out real test sets. Synthetic data were generated only from the training data and combined with the real training data for model fitting. Final model performance was evaluated only on held-out real test data. This workflow provides internal validation and does not replace external, cross-site, prospective, or longitudinal validation.

The prediction model performance was evaluated using Accuracy (ACC), F1 score, and Area Under the Receiver Operating Characteristic Curve (AUC), all of which are widely used in the assessment of health prediction models. To evaluate the model explanation quality, we used two key metrics. The Spearman’s rank correlation coefficient was used to assess global explanation stability by comparing feature importance rankings obtained from the explainable model trained on the original dataset with those trained on augmented datasets using different data augmentation strategies at varying augmentation ratios [54]. To quantify local explanation stability, we adopted an input perturbation approach and used cosine similarity, a widely used metric in perturbation-based explanation evaluation, to compute the similarity between the local additive contribution vectors of the original and perturbed input instances across multiple runs [55]. Perturbed samples were created by applying low-probability flips to features (flip probability = 0.05–0.08), ensuring that explanations remained relevant. Thirty instances were randomly selected for evaluation, and the final stability score was computed as the mean cosine similarity across these instances over 10 runs.

## 4 Results

### 4.1 Prediction model performance

The performances of GC, CTGAN, and TVAE (Tables 2, 3, and 4) in generating synthetic data to augment the training dataset were evaluated alongside five machine learning models: SVM, RF, GB, LR, and EBM, for predicting perinatal psychological distress among Aboriginal mothers using the adopted dataset. The models were trained on the original dataset (augmented data ratio = 0) and on augmented training datasets with ratios ranging from 0.25 to 2. Mean accuracy, F1 score, and AUC, along with their standard deviations, were reported.

**Table 2:** Performance comparison of GC data augmentation techniques with varying data augmentation ratios using different machine learning methods.

Model	Ratio	ACC	F1 Score	AUC
GC + SVM	0	0.819 (± 0.008)	0.703 (± 0.013)	0.876 (± 0.032)
	0.25	0.824 (± 0.003)	0.704 (± 0.001)	0.871 (± 0.003)
	0.5	0.819 (± 0.074)	0.686 (± 0.001)	0.868 (± 0.037)
	0.75	0.807 (± 0.037)	0.669 (± 0.007)	0.862 (± 0.074)
	1	0.807 (± 0.074)	0.673 (± 0.060)	0.856 (± 0.051)
	1.25	0.819 (± 0.084)	0.689 (± 0.070)	0.863 (± 0.065)
	1.5	0.819 (± 0.037)	0.686 (± 0.107)	0.863 (± 0.011)
	1.75	0.819 (± 0.037)	0.691 (± 0.131)	0.856 (± 0.100)
	2	0.813 (± 0.074)	0.680 (± 0.106)	0.855 (± 0.037)
GC + RF	0	0.836 (± 0.032)	0.771 (± 0.047)	0.918 (± 0.010)
	0.25	0.841 (± 0.074)	0.783 (± 0.033)	0.918 (± 0.011)
	0.5	0.853 (± 0.074)	0.790 (± 0.037)	0.904 (± 0.011)
	0.75	0.774 (± 0.037)	0.706 (± 0.014)	0.881 (± 0.037)
	1	0.813 (± 0.037)	0.752 (± 0.015)	0.901 (± 0.074)
	1.25	0.796 (± 0.037)	0.709 (± 0.052)	0.878 (± 0.037)
	1.5	0.807 (± 0.037)	0.723 (± 0.092)	0.866 (± 0.037)
	1.75	0.785 (± 0.039)	0.703 (± 0.048)	0.865 (± 0.037)
	2	0.824 (± 0.074)	0.763 (± 0.037)	0.889 (± 0.047)
GC + GB	0	0.825 (± 0.040)	0.726 (± 0.062)	0.929 (± 0.016)
	0.25	0.819 (± 0.037)	0.711 (± 0.033)	0.923 (± 0.074)
	0.5	0.824 (± 0.037)	0.719 (± 0.031)	0.919 (± 0.037)
	0.75	0.802 (± 0.037)	0.676 (± 0.070)	0.905 (± 0.074)
	1	0.819 (± 0.074)	0.711 (± 0.028)	0.908 (± 0.029)
	1.25	0.813 (± 0.037)	0.689 (± 0.053)	0.907 (± 0.037)
	1.5	0.813 (± 0.037)	0.710 (± 0.053)	0.909 (± 0.037)
	1.75	0.836 (± 0.011)	0.741 (± 0.075)	0.916 (± 0.037)
	2	0.847 (± 0.011)	0.764 (± 0.058)	0.911 (± 0.054)
GC + LR	0	0.797 (± 0.037)	0.697 (± 0.083)	0.851 (± 0.006)
	0.25	0.779 (± 0.045)	0.671 (± 0.064)	0.837 (± 0.037)
	0.5	0.790 (± 0.043)	0.690 (± 0.044)	0.822 (± 0.015)
	0.75	0.774 (± 0.034)	0.667 (± 0.040)	0.826 (± 0.074)
	1	0.785 (± 0.037)	0.682 (± 0.050)	0.829 (± 0.014)
	1.25	0.785 (± 0.059)	0.681 (± 0.050)	0.821 (± 0.037)
	1.5	0.774 (± 0.037)	0.659 (± 0.047)	0.829 (± 0.037)
	1.75	0.779 (± 0.037)	0.668 (± 0.034)	0.827 (± 0.037)
	2	0.762 (± 0.085)	0.647 (± 0.042)	0.818 (± 0.037)
GC + EBM	0	0.836 (± 0.032)	0.752 (± 0.048)	0.933 (± 0.016)
	0.25	0.796 (± 0.037)	0.666 (± 0.016)	0.923 (± 0.025)
	0.5	0.802 (± 0.074)	0.673 (± 0.044)	0.916 (± 0.074)
	0.75	0.796 (± 0.037)	0.667 (± 0.031)	0.914 (± 0.037)
	1	0.813 (± 0.037)	0.691 (± 0.035)	0.911 (± 0.050)
	1.25	0.819 (± 0.074)	0.703 (± 0.009)	0.911 (± 0.074)
	1.5	0.824 (± 0.037)	0.719 (± 0.037)	0.911 (± 0.074)
	1.75	0.819 (± 0.074)	0.712 (± 0.053)	0.910 (± 0.074)
	2	0.802 (± 0.051)	0.679 (± 0.047)	0.901 (± 0.037)

GC: Gaussian Copula, SVM: Support Vector Machine, RF: Random Forest, GB: Gradient Boosting Machine, LR: Logistic Regression, EBM: Explainable Boosting Machine.

**Table 3:** Performance comparison of CTGAN data augmentation techniques with varying data augmentation ratios using different machine learning methods.

Model	Ratio	ACC	F1 Score	AUC
CTGAN+SVM	0	0.819 (± 0.008)	0.703 (± 0.013)	0.876 (± 0.032)
	0.25	0.820 (± 0.013)	0.701 (± 0.019)	0.875 (± 0.005)
	0.5	0.816 (± 0.014)	0.700 (± 0.032)	0.876 (± 0.009)
	0.75	0.818 (± 0.028)	0.705 (± 0.053)	0.866 (± 0.010)
	1	0.814 (± 0.031)	0.685 (± 0.058)	0.864 (± 0.011)
	1.25	0.795 (± 0.031)	0.641 (± 0.086)	0.866 (± 0.017)
	1.5	0.810 (± 0.024)	0.679 (± 0.068)	0.870 (± 0.011)
	1.75	0.789 (± 0.035)	0.630 (± 0.091)	0.872 (± 0.017)
	2	0.798 (± 0.017)	0.643 (± 0.055)	0.870 (± 0.022)
CTGAN+RF	0	0.836 (± 0.032)	0.771 (± 0.047)	0.918 (± 0.010)
	0.25	0.826 (± 0.026)	0.765 (± 0.037)	0.910 (± 0.014)
	0.5	0.818 (± 0.028)	0.757 (± 0.040)	0.897 (± 0.015)
	0.75	0.800 (± 0.023)	0.734 (± 0.029)	0.884 (± 0.016)
	1	0.800 (± 0.029)	0.736 (± 0.041)	0.889 (± 0.024)
	1.25	0.806 (± 0.039)	0.737 (± 0.055)	0.889 (± 0.028)
	1.5	0.779 (± 0.025)	0.699 (± 0.033)	0.871 (± 0.020)
	1.75	0.803 (± 0.027)	0.734 (± 0.037)	0.872 (± 0.023)
	2	0.798 (± 0.034)	0.730 (± 0.046)	0.885 (± 0.034)
CTGAN+GB	0	0.825 (± 0.040)	0.726 (± 0.062)	0.929 (± 0.016)
	0.25	0.846 (± 0.023)	0.762 (± 0.032)	0.921 (± 0.009)
	0.5	0.846 (± 0.018)	0.766 (± 0.032)	0.931 (± 0.010)
	0.75	0.831 (± 0.016)	0.743 (± 0.028)	0.916 (± 0.010)
	1	0.825 (± 0.025)	0.729 (± 0.035)	0.916 (± 0.013)
	1.25	0.811 (± 0.023)	0.695 (± 0.047)	0.918 (± 0.021)
	1.5	0.818 (± 0.026)	0.705 (± 0.051)	0.910 (± 0.016)
	1.75	0.815 (± 0.021)	0.695 (± 0.046)	0.912 (± 0.016)
	2	0.818 (± 0.024)	0.702 (± 0.062)	0.917 (± 0.016)
CTGAN+LR	0	0.797 (± 0.037)	0.697 (± 0.083)	0.851 (± 0.006)
	0.25	0.784 (± 0.015)	0.682 (± 0.030)	0.854 (± 0.006)
	0.5	0.788 (± 0.020)	0.697 (± 0.033)	0.850 (± 0.010)
	0.75	0.786 (± 0.023)	0.696 (± 0.033)	0.849 (± 0.010)
	1	0.782 (± 0.018)	0.686 (± 0.025)	0.843 (± 0.012)
	1.25	0.759 (± 0.019)	0.654 (± 0.055)	0.833 (± 0.015)
	1.5	0.788 (± 0.028)	0.688 (± 0.060)	0.838 (± 0.015)
	1.75	0.785 (± 0.031)	0.680 (± 0.061)	0.837 (± 0.019)
	2	0.793 (± 0.017)	0.689 (± 0.069)	0.844 (± 0.014)
CTGAN+EBM	0	0.836 (± 0.032)	0.752 (± 0.048)	0.933 (± 0.016)
	0.25	0.844 (± 0.013)	0.754 (± 0.025)	0.933 (± 0.007)
	0.5	**0.853 (± 0.023)**	**0.770 (± 0.048)**	**0.936 (± 0.007)**
	0.75	0.837 (± 0.037)	0.752 (± 0.068)	0.920 (± 0.012)
	1	0.836 (± 0.020)	0.746 (± 0.043)	0.919 (± 0.011)
	1.25	0.809 (± 0.023)	0.684 (± 0.057)	0.911 (± 0.014)
	1.5	0.811 (± 0.029)	0.676 (± 0.078)	0.909 (± 0.011)
	1.75	0.807 (± 0.020)	0.678 (± 0.064)	0.909 (± 0.018)
	2	0.801 (± 0.033)	0.645 (± 0.111)	0.916 (± 0.020)

CTGAN: Conditional Tabular Generative Adversarial Network, SVM: Support Vector Machine, RF: Random Forest, GB: Gradient Boosting Machine, LR: Logistic Regression, EBM: Explainable Boosting Machine.

**Table 4:** Performance comparison of TVAE data augmentation techniques with varying data augmentation ratios using different machine learning methods.

Models	Ratio	ACC	F1 Score	AUC
TVAE+SVM	0	0.819 (± 0.008)	0.703 (± 0.013)	0.876 (± 0.032)
	0.25	0.817 (± 0.014)	0.717 (± 0.027)	0.871 (± 0.006)
	0.5	0.816 (± 0.012)	0.730 (± 0.019)	0.863 (± 0.005)
	0.75	0.819 (± 0.010)	0.741 (± 0.017)	0.860 (± 0.008)
	1	0.812 (± 0.011)	0.737 (± 0.017)	0.860 (± 0.006)
	1.25	0.809 (± 0.017)	0.741 (± 0.021)	0.854 (± 0.010)
	1.5	0.811 (± 0.017)	0.745 (± 0.020)	0.857 (± 0.007)
	1.75	0.815 (± 0.017)	0.751 (± 0.024)	0.855 (± 0.011)
	2	0.801 (± 0.019)	0.737 (± 0.021)	0.852 (± 0.012)
TVAE+RF	0	0.836 (± 0.032)	0.771 (± 0.047)	0.918 (± 0.010)
	0.25	0.806 (± 0.019)	0.747 (± 0.023)	0.908 (± 0.010)
	0.5	0.799 (± 0.022)	0.745 (± 0.023)	0.901 (± 0.013)
	0.75	0.774 (± 0.032)	0.723 (± 0.034)	0.896 (± 0.014)
	1	0.781 (± 0.026)	0.734 (± 0.028)	0.899 (± 0.012)
	1.25	0.782 (± 0.021)	0.745 (± 0.022)	0.895 (± 0.010)
	1.5	0.751 (± 0.027)	0.707 (± 0.027)	0.893 (± 0.012)
	1.75	0.767 (± 0.025)	0.722 (± 0.023)	0.883 (± 0.010)
	2	0.765 (± 0.032)	0.724 (± 0.030)	0.888 (± 0.011)
TVAE+GB	0	0.825 (± 0.040)	0.726 (± 0.062)	0.929 (± 0.016)
	0.25	0.824 (± 0.020)	0.734 (± 0.039)	0.928 (± 0.004)
	0.5	0.832 (± 0.013)	0.748 (± 0.024)	0.927 (± 0.005)
	0.75	0.832 (± 0.017)	0.751 (± 0.030)	0.931 (± 0.007)
	1	0.846 (± 0.013)	0.774 (± 0.018)	0.928 (± 0.009)
	1.25	0.835 (± 0.014)	0.759 (± 0.025)	0.926 (± 0.008)
	1.5	0.842 (± 0.012)	0.772 (± 0.019)	0.925 (± 0.007)
	1.75	0.837 (± 0.014)	0.765 (± 0.019)	0.926 (± 0.005)
	2	0.838 (± 0.015)	0.768 (± 0.019)	0.923 (± 0.006)
TVAE+LR	0	0.797 (± 0.037)	0.697 (± 0.083)	0.851 (± 0.006)
	0.25	0.793 (± 0.013)	0.711 (± 0.022)	0.852 (± 0.006)
	0.5	0.794 (± 0.017)	0.716 (± 0.025)	0.853 (± 0.006)
	0.75	0.803 (± 0.011)	0.729 (± 0.011)	0.852 (± 0.006)
	1	0.792 (± 0.019)	0.716 (± 0.021)	0.850 (± 0.008)
	1.25	0.795 (± 0.016)	0.723 (± 0.022)	0.848 (± 0.007)
	1.5	0.790 (± 0.017)	0.715 (± 0.025)	0.845 (± 0.007)
	1.75	0.790 (± 0.017)	0.721 (± 0.023)	0.845 (± 0.005)
	2	0.790 (± 0.013)	0.721 (± 0.018)	0.844 (± 0.008)
TVAE+EBM	0	0.836 (± 0.032)	0.752 (± 0.048)	0.933 (± 0.016)
	0.25	0.863 (± 0.010)	0.806 (± 0.014)	0.935 (± 0.005)
	0.5	0.864 (± 0.012)	0.813 (± 0.018)	0.937 (± 0.005)
	0.75	0.864 (± 0.018)	0.816 (± 0.025)	0.933 (± 0.007)
	1	**0.867 (± 0.015)**	**0.817 (± 0.019)**	**0.934 (± 0.007)**
	1.25	0.862 (± 0.014)	0.812 (± 0.020)	0.931 (± 0.005)
	1.5	0.864 (± 0.017)	0.816 (± 0.024)	0.931 (± 0.006)
	1.75	0.849 (± 0.013)	0.796 (± 0.021)	0.932 (± 0.005)
	2	0.860 (± 0.012)	0.810 (± 0.017)	0.929 (± 0.007)

TVAE: Tabular Variational Autoencoder, SVM: Support Vector Machine, RF: Random Forest, GB: Gradient Boosting Machine, LR: Logistic Regression, EBM: Explainable Boosting Machine.

The EBM classifier demonstrated the best overall internal performance among the five models. Without data augmentation, it achieved an accuracy of 0.836, an F1 score of 0.752, and an AUC of 0.933. With GC augmentation, the performance of EBM remained lower than that of the EBM model trained on the original dataset. With CTGAN augmentation, EBM’s performance improved as the augmented data ratio increased from 0.25 to 0.5, but declined as the ratio increased further. The CTGAN-augmented EBM achieved its highest performance at a ratio of 0.5, with an accuracy of 0.853, an F1 score of 0.770, and an AUC of 0.936. For TVAE, data augmentation also improved the internal performance of the EBM model. The highest performance was observed at an augmentation ratio of 1.0, with an accuracy of 0.867, an F1 score of 0.817, and an AUC of 0.934 on held-out real test data. Across the various augmentation ratios, the model generally demonstrated improvements in accuracy and F1 score compared with using the original training data alone. These findings suggest that TVAE-based augmentation may improve model learning for this pilot dataset.

We also found that GB, another ensemble learning method, achieved the second-best overall performance. GC + GB showed improved performance when the augmented data ratio ranged from 1.75 to 2 compared to training on the original dataset. Similarly, CTGAN+GB showed improved performance when the augmented data ratio ranged from 0.25 to 0.75 compared to training on the original data alone, achieving the best results at a ratio of 0.5, with an accuracy of 0.846, an F1 score of 0.766, and an AUC of 0.931. Also, TVAE+GB exhibited an enhanced performance as the augmented data ratio increased from 0.5 to 2, achieving its highest accuracy of 0.846 and an F1 score of 0.774 at a ratio of 1.0.

However, compared to EBM, GB consistently underperformed, regardless of whether GC, CTGAN, or TVAE was used. For the remaining models, including SVM, RF, and LR, no consistent performance improvements were observed with either augmentation technique.

Figs 2, 3, and 4 illustrate the internal F1 score performance across varying augmented data ratios using GC, CTGAN, and TVAE synthesizers, respectively. For CTGAN, most models, except for RF, achieved better internal prediction performance at a ratio of 0.5. For TVAE, the optimal data augmentation ratio was between 0.75 and 1.0 for most models, again with the exception of RF. Further increasing the augmented data ratio beyond 1.0 did not improve internal test performance.

**Fig 2:**
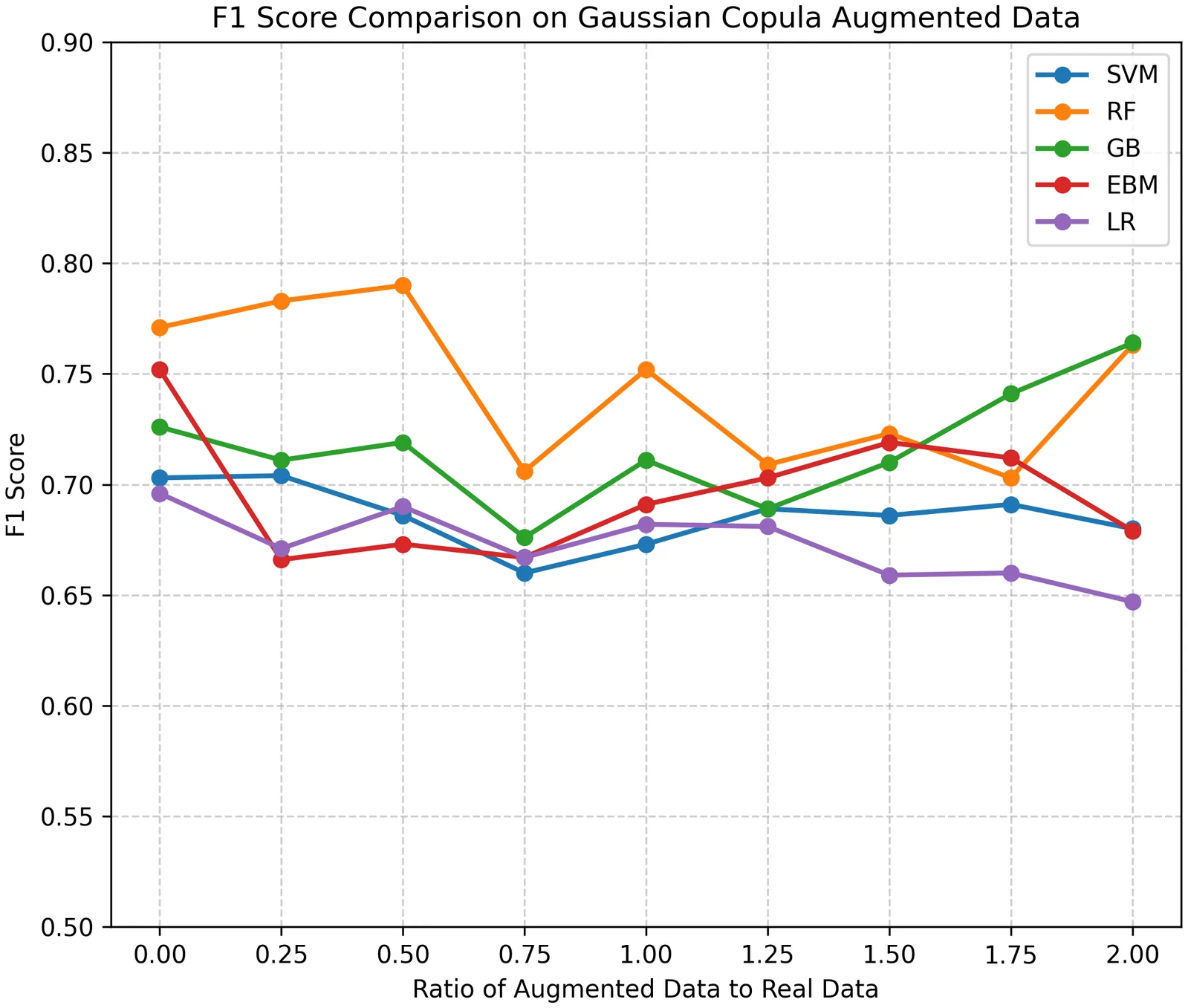
Internal F1 score across Gaussian Copula augmentation ratios. Ratio 0 represents models trained on the original training data without synthetic augmentation. Points show mean F1 scores across repeated experimental cycles and synthetic-data variations.

**Fig 3:**
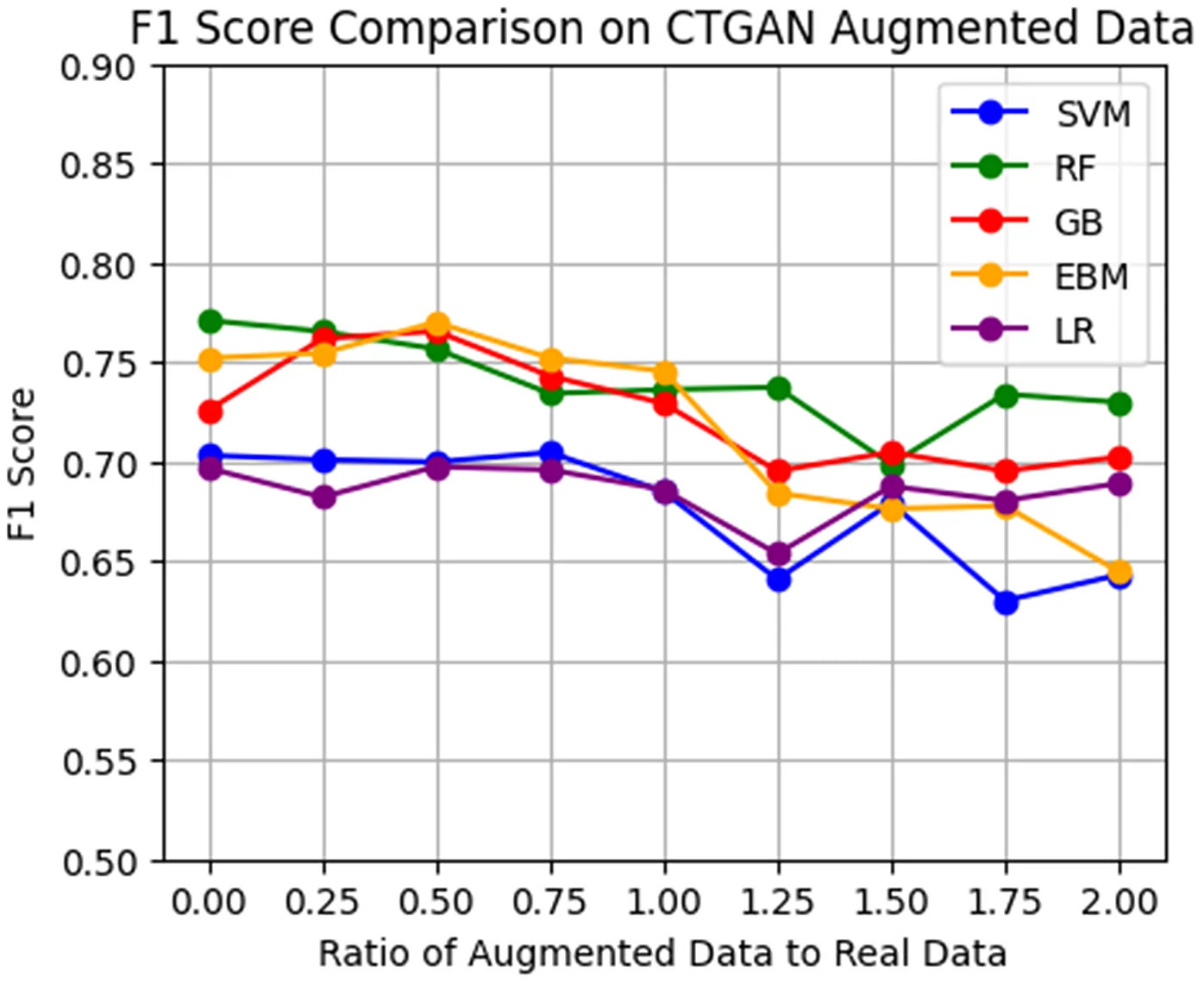
Internal F1 score across CTGAN augmentation ratios. Ratio 0 represents models trained on the original training data without synthetic augmentation. Points show mean F1 scores across repeated experimental cycles and synthetic-data variations.

**Fig 4:**
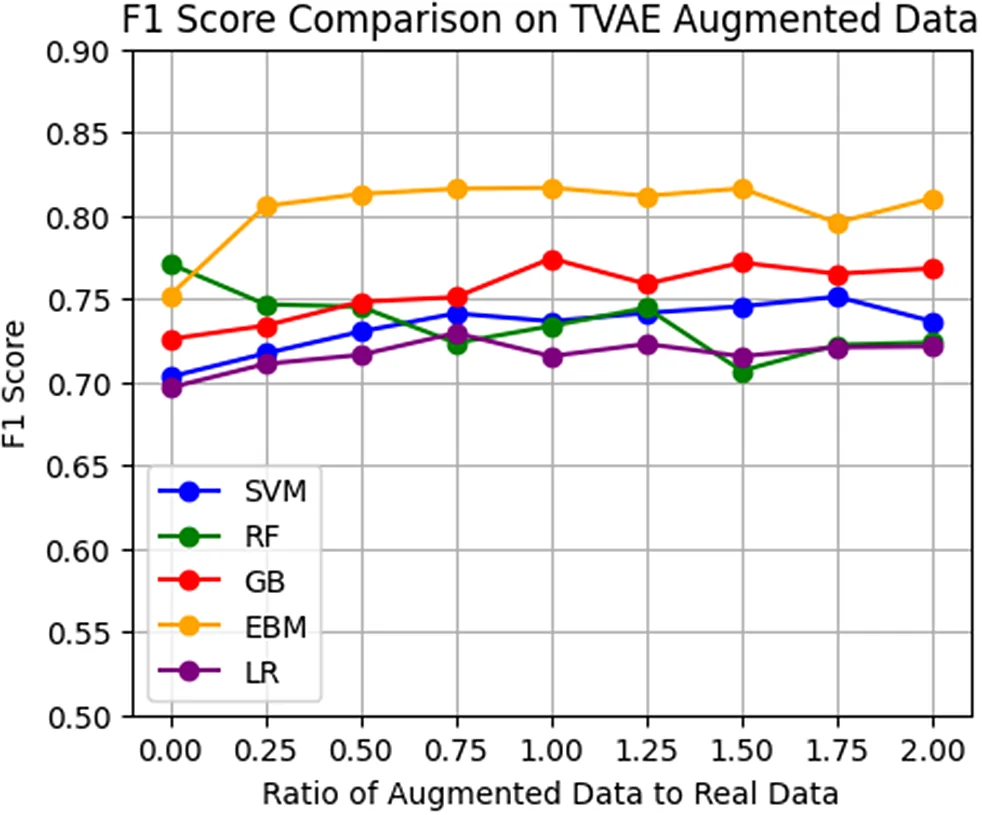
Internal F1 score across TVAE augmentation ratios. Ratio 0 represents models trained on the original training data without synthetic augmentation. Points show mean F1 scores across repeated experimental cycles and synthetic-data variations. TVAE+EBM at a ratio of 1.0 achieved the highest internal F1 score among evaluated configurations.

Thus, it is important to carefully design the generative AI techniques alongside the machine learning models, particularly when determining key parameter settings such as the augmented data ratio, to ensure that the constructed model’s performance for the learning task is optimal. In summary, TVAE+EBM with an augmentation ratio of 1.0 achieved the best internal performance among the evaluated configurations and was selected as the primary exploratory model for interpretation.

### 4.2 Feature importance agreement and explanation stability under augmentation

#### 4.2.1 Global explanation stability under data augmentation.

Table 5 presents the Spearman’s rank correlation, which was used to compare feature-importance rankings learned from EBMs trained on the original dataset with those trained on augmented datasets generated using GC, CTGAN, and TVAE across multiple augmentation ratios. Across all augmentation ratios, correlations remained positive, indicating that data augmentation generally preserved the relative ranking of influential predictors. CTGAN+EBM achieved the highest single Spearman correlation at an augmentation ratio of 1.0, with a value of 0.890, but its values varied substantially across ratios, ranging from 0.448 to 0.890. In contrast, TVAE+EBM demonstrated more consistent global explanation agreement across ratios, with Spearman correlations ranging from 0.734 to 0.822. The GC-based augmentation method showed intermediate behaviour, with values ranging from 0.520 to 0.766. Together with its superior internal predictive performance and local explanation stability, this supported the selection of TVAE+EBM at an augmentation ratio of 1.0 as the primary exploratory model.

**Table 5:** Global explanation stability in terms of Spearman’s rank correlation computed between feature importance rankings learned from EBM trained on the original dataset and augmented data under different augmentation ratios.

Models	Spearman’s rank correlation							
	Augmentation ratio							
	0.25	0.5	0.75	1.0	1.25	1.5	1.75	2.0
GC + EBM	0.766	0.760	0.673	0.655	0.657	0.520	0.562	0.663
CTGAN + EBM	0.696	0.655	0.679	0.890	0.473	0.448	0.566	0.548
TVAE + EBM	0.808	0.734	0.783	0.822	0.779	0.747	0.757	0.734

#### 4.2.2 Local explanation stability under data augmentation.

Table 6 summarises the cosine similarity values computed using an input perturbation-based approach across multiple augmentation ratios and different synthesizers. Stability remained consistently high across synthetic data methods and augmentation ratios, with average cosine similarity values generally exceeding 0.93. Among the evaluated augmentation methods, TVAE+EBM showed the highest local explanation stability across augmentation ratios, with the highest cosine similarity observed at *r* = 1.0. GC + EBM and CTGAN+EBM also showed high local stability, but with slightly lower values overall. These results suggest that local explanations from TVAE+EBM were relatively stable under small input perturbations within this internal evaluation setting.

**Table 6:** Local explanation stability of EBM measured by cosine similarity between additive feature contribution vectors of original and perturbed input instances under different augmentation ratios.

Models	Cosine similarity							
	Augmentation ratio							
	0.25	0.5	0.75	1.0	1.25	1.5	1.75	2.0
GC + EBM	0.936	0.933	0.929	0.941	0.942	0.932	0.946	0.929
CTGAN+EBM	0.939	0.933	0.945	0.945	0.938	0.934	0.946	0.947
TVAE+EBM	0.950	0.935	0.953	0.968	0.958	0.955	0.950	0.957

### 4.3 Model explanation interpretation

We created a prototype interactive dashboard to display explanations from the best-performing internally evaluated EBM. In the visualisation, we incorporated culturally relevant images related to the BCYR questions and responses, and highlighted important features selected by the EBM to enhance visualisation and user understanding. The explanations focus on two aspects: global explanations, which examine overall feature importance levels across the dataset; and local explanations, which highlight key contributing features and their weightings influencing the prediction for each individual mother.

The following explanations describe how the trained EBM model used the selected variables to generate predictions in the study dataset. They indicate which responses were most influential for this internally validated model and may help guide further clinical and community interpretation.

#### 4.3.1 Global explanation.

We generated explanations from the EBM and illustrated global feature importance with quantitative weightings across the study dataset in Fig 5. These values indicate how strongly each feature contributed to the model’s predictions, rather than causal effects. For instance, the feature “Blaming Herself” with a weighting of 0.70 emerged as the most influential feature in the model, suggesting that responses to this question about self-blame were strongly associated with model-estimated perinatal psychological distress in this dataset. This was followed by features such as “Feeling Lonely” with a weighting of 0.44 and “Makes Family Proud” with a weighting of 0.35. Other features, including “Managing Day-to-Day” with a weighting of 0.34, “Trouble Sleeping” with a weighting of 0.32, and “Life Not Worth Living” with a weighting of 0.27, also contributed to outcome prediction. The remaining influential features and their weightings are shown in Fig 5.

**Fig 5:**
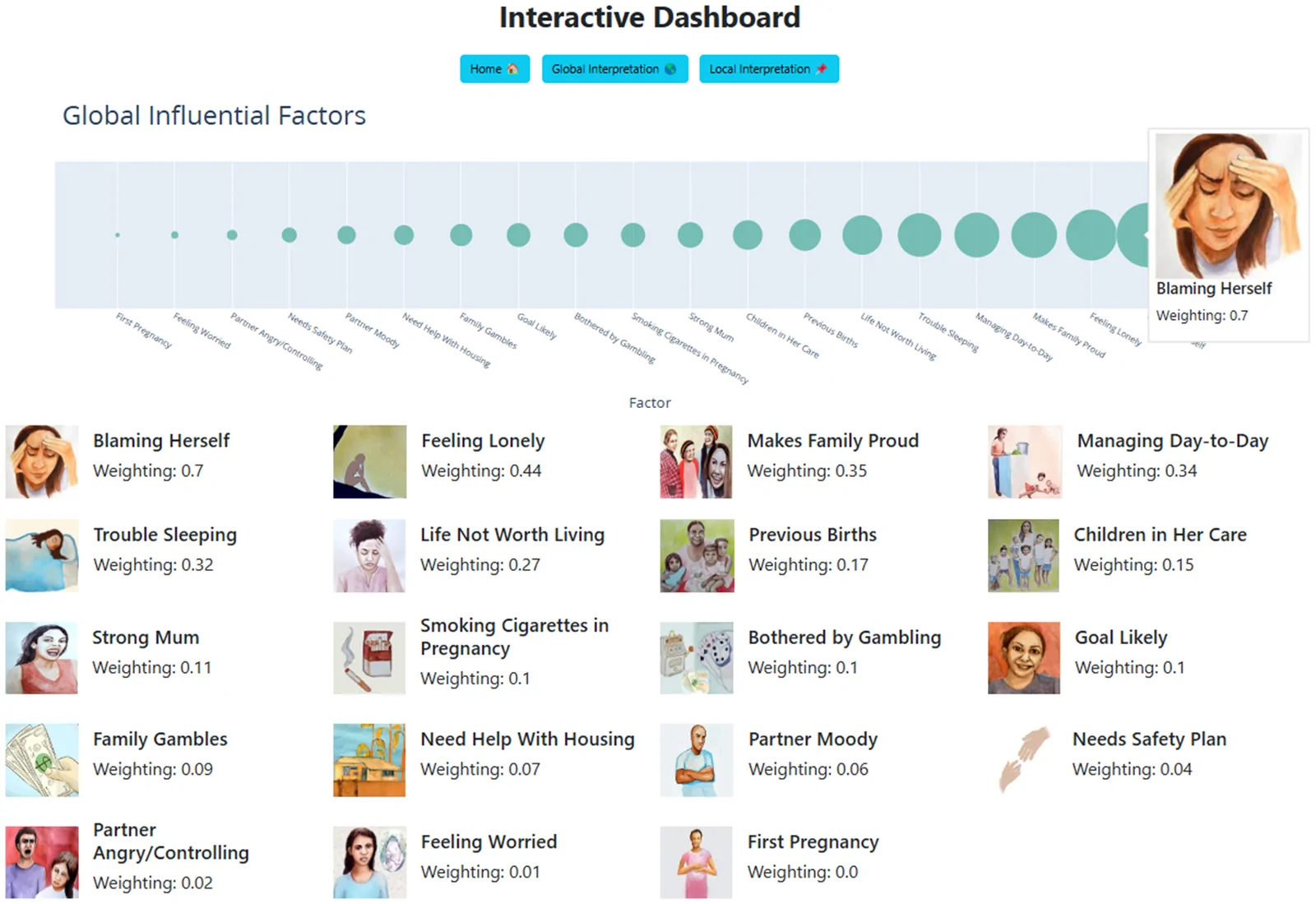
Prototype dashboard for global EBM feature importance. The plot summarises variables that contributed most strongly to model predictions across the study dataset.

#### 4.3.2 Local explanation.

The EBM model also provides interpretations for individual predictions, enabling deeper insight into model-estimated risk. Fig 6 presents an example case of an Aboriginal woman classified by the model as low risk. The visualisation shows the specific feature contributions that shifted the prediction toward or away from the low-risk category for this individual. The y-axis of the plot displays the most relevant features, with the corresponding responses from this individual mother labelled on the horizontal bars.

**Fig 6:**
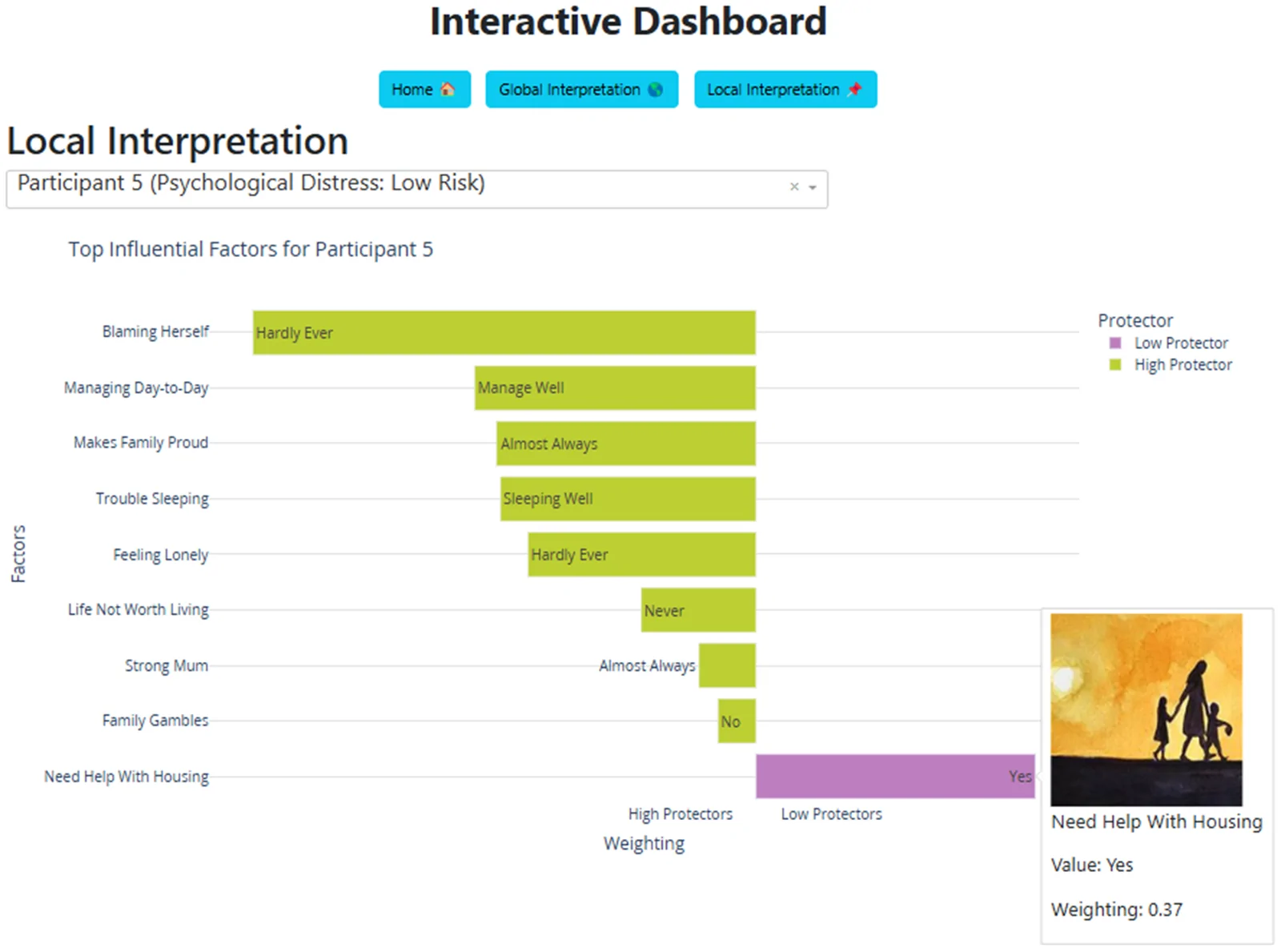
Prototype local explanation dashboard for one low-risk case. Green bars indicate responses that shifted the model prediction toward lower estimated risk, while purple bars indicate responses that shifted the prediction toward higher estimated risk.

Responses that shifted the model-estimated prediction toward the low-risk category are highlighted in green. These include responses such as “Blaming Herself” = Hardly Ever, “Managing Day-to-Day” = Manage Well, “Makes Family Proud” = Almost Always, “Trouble Sleeping” = Sleeping Well, and “Feeling Lonely” = Hardly Ever.

Conversely, features displayed in purple shifted the model’s prediction away from the low-risk category. For example, “Need Help With Housing” = Yes increased the model-estimated risk for this individual. In this example, the cumulative influence of responses shifting the prediction toward lower estimated risk outweighed those shifting it toward higher estimated risk.

## 5 Discussion

In summary, the TVAE+EBM-based XAI model demonstrated the highest internal predictive performance among the evaluated configurations while also offering inherent interpretability. The model provides human-readable explanations of how selected variables contributed to predictions in this dataset; however, these explanations should not be interpreted as causal evidence or as proof of clinical effectiveness.

The global feature-importance analysis identified several variables that contributed substantially to model-estimated perinatal psychological distress. Among these, “Blaming Herself” emerged as the most influential feature, suggesting that expressions of self-blame play an important role in the model’s predictions. This finding may point to the importance of healthcare workers attending to expressions of self-blame in clinical interactions, as these may present opportunities to initiate supportive conversations and follow-ups and to strengthen Aboriginal women’s perinatal self-efficacy and resilience, which are central to social and emotional well-being.

Other highly influential features, such as “Feeling Lonely” and “Makes Family Proud,” further highlight the importance of social and relational dimensions within the model. The prominence of these features is consistent with existing literature emphasising the role of social connectedness and family relationships in perinatal mental health among Aboriginal women [11,56]. While global analyses identify these variables as influential predictors without indicating causality, local interpretability analyses provide additional insight into how individual feature values may function as risk-related or protective-related factors within model outputs.

Importantly, this commitment to XAI and to making complex data accessible and understandable is a guiding principle of our research team. We have previously emphasised the importance of privileging Indigenous stories as data, as well as the critical need to present complex data in narrative form, thereby valuing cultural knowledge and establishing a foundational requirement: that Indigenous participants must be able to interpret and benefit from the findings of our work [57]. This approach also reflects our continued commitment to addressing long-standing inequities in perinatal mental health assessment for Indigenous women [58–61]. This level of transparency may support health professionals in understanding why the model generated a particular estimate and may help structure culturally safe conversations. However, explainability is a necessary but insufficient condition for safe clinical use. Transparent explanations do not, by themselves, establish clinical usefulness, fairness, safety, cultural acceptability, or improved outcomes. Whether the dashboard improves communication, trust, referral appropriateness, or maternal and infant outcomes requires future prospective evaluation and ongoing co-design with Aboriginal women, Elders, communities, and health professionals.

### 5.1 Bias amplification and fairness considerations

Bias amplification is an important risk when applying generative AI to small health datasets. Because synthetic data generation learns from the observed dataset, it may reproduce or intensify existing biases, including site-level recruitment patterns, underrepresentation of particular communities or experiences, missingness, nonresponse, and social desirability bias. In the context of Aboriginal perinatal mental health, such biases could have important consequences if model outputs were interpreted without appropriate cultural and clinical context.

The present study sought to reduce this risk by fitting synthesizers only on training data and evaluating models on held-out real data. However, the available sample was too small to support robust subgroup fairness analysis across relevant dimensions, such as age, geography, language group, parity, and community context. Site identifiers were not available in the analytic dataset, which also prevented cross-site testing or leave-one-site-out validation. Future work should assess predictive discrimination, calibration, and potential clinical consequences across relevant groups using community-governed protocols.

### 5.2 Ethical AI use, Indigenous data governance, and community control

Ethical AI use in this context must be grounded in Aboriginal leadership, data sovereignty, community benefit, and ongoing accountability. The BCYR program was co-designed with Aboriginal women, Elders, Aboriginal researchers, midwives, and health professionals. This study used only deidentified data from participants who had consented to research use. Model development, interpretation, dashboard design, data access, and any future implementation should remain subject to Aboriginal-led governance and community oversight. Synthetic data should not be treated as a substitute for consent, data sovereignty, or community control, and should not be used to bypass restrictions on culturally sensitive data. Any future use or deployment should be assessed against principles of collective benefit, authority to control, responsibility, and ethics, and should align with national guidance for ethical Aboriginal and Torres Strait Islander research.

The model should also operate strictly as a human-in-the-loop decision-support tool. Model outputs should not be used to make automated decisions, replace clinical judgment, determine eligibility for care, trigger punitive responses, or override Aboriginal women’s own priorities and self-determined needs. Instead, model explanations should support strengths-based, culturally safe conversations between mothers and trusted health professionals.

### 5.3 Proposed clinical workflow and implementation implications

If future validation supports implementation, the dashboard would be embedded within the existing BCYR workflow rather than used as a separate screening instrument. After an Aboriginal mother completes the BCYR assessment, the model would generate prediction outputs and a visual explanation of the most influential strengths and worries for that individual. The clinician would review this output as a conversation aid, not as a diagnosis. The intended workflow is: first, the mother completes the BCYR assessment in the usual culturally adapted format; second, the dashboard displays protective-related and risk-related factors using accessible language and BCYR visual elements; third, the midwife or clinician discusses these key factors with the mother, confirms whether the explanation reflects her lived experience, and invites her to identify priorities and supports; fourth, the clinician combines this discussion with clinical judgment, local referral pathways, safety protocols, and the mother’s preferences; and fifth, the service documents actions taken and monitors whether the tool supports engagement, trust, and timely care.

This workflow has implementation implications. Clinicians would require training in culturally safe interpretation of model outputs, limitations of AI, strengths-based communication, and avoidance of deterministic or punitive use. Services would also need clear governance regarding documentation, accountability, escalation pathways, audit, and processes for Aboriginal women and communities to provide feedback or challenge harmful interpretations.

## 6 Limitations

This study has several important limitations. First, the model was developed using a pilot dataset of 288 participants after outcome-based exclusions. Although repeated internal validation, cross-validation, and synthetic augmentation were used to reduce instability, these methods cannot substitute for a larger and more representative real-world dataset. Second, participants were drawn from a convenience sample across BCYR pilot sites. The sample may not represent the full diversity of Aboriginal mothers across Western Australia or other regions, including differences in language, Country, community context, age, parity, rurality, service access, and local models of care. External validation is therefore needed in the future. Third, synthetic data can reproduce or amplify biases present in the source data, particularly when the original dataset is small or does not capture the full diversity of relevant communities, experiences, and service contexts. For this reason, synthetic data were used only for training augmentation, and all model evaluation was performed on held-out real data. Fourth, the study did not include independent external validation, prospective validation, cross-site deployment testing, or longitudinal assessment. Site identifiers do not exist in the analytic dataset. Caution is warranted when interpreting the model’s generalisability to other services, time periods, or communities, as well as its capacity to improve maternal or infant outcomes through model-supported care. Fifth, assessment responses may be affected by nonresponse, social desirability bias, fear of judgment, and variation in trust or cultural safety across services. These factors may influence both the original assessment data and the model outputs. Explainable AI can help identify influential variables, but it cannot fully detect or correct these biases. Finally, the dashboard remains an early prototype. Its cultural meaning, usability, acceptability, clinical workflow fit, and potential unintended consequences require further Aboriginal-led co-design and evaluation before any clinical implementation. Future research should broaden the dataset, conduct external and cross-site validation when suitable data and site identifiers are available, assess calibration and fairness, evaluate longitudinal outcomes, and refine the dashboard through community-governed implementation research.

## 7 Conclusion

This exploratory study examined the use of generative AI techniques, specifically CTGAN and TVAE, combined with the glassbox model EBM, to predict perinatal psychological distress among Aboriginal mothers using BCYR pilot data. The results suggest that TVAE-based data augmentation can improve internal EBM performance in this dataset while maintaining stable model explanations. Furthermore, the glassbox nature of EBM enabled the model to provide transparent explanations for its predictions, offering insight into the AI’s decision-making process. These explanations should be treated as model-generated prompts for culturally safe discussion rather than as clinical diagnoses or automated decisions.

The involvement of Aboriginal women with lived experience and Aboriginal and health researchers guided the co-design of culturally relevant assessment items, helping ensure that the learned models and their predictions reflected perspectives grounded in cultural knowledge and community needs. Future research should further assess the cultural appropriateness of AI-generated explanations to strengthen trust and usability in clinical practice. This exploratory study suggests that interpretable, generative AI-assisted modelling may have potential to support culturally responsive perinatal mental health assessment. However, the current model should not be considered clinically validated or implementation-ready. Further Aboriginal-led co-design, independent external validation, prospective workflow evaluation, fairness auditing, and longitudinal outcome assessment are required before any clinical use. This work represents the beginning of a broader journey to use innovative approaches to improve healthcare assessment and outcomes for Aboriginal mothers. We hope it will encourage further research and discussion about the transparent use of AI technologies in Indigenous contexts. We also invite engagement with other groups working to develop culturally safe and sound AI tools and techniques.
